# Sono-Photocatalytic Activity of Cloisite 30B/ZnO/Ag_2_O Nanocomposite for the Simultaneous Degradation of Crystal Violet and Methylene Blue Dyes in Aqueous Media

**DOI:** 10.3390/nano12183103

**Published:** 2022-09-07

**Authors:** Rauf Foroutan, Seyed Jamaleddin Peighambardoust, Daria Camilla Boffito, Bahman Ramavandi

**Affiliations:** 1 Faculty of Chemical and Petroleum Engineering, University of Tabriz, Tabriz 5166616471, Iran; 2 Department of Chemical Engineering, Polytechnique Monteral, Monteral, QC H3C 3A7, Canada; 3 Systems Environmental Health and Energy Research Center, The Persian Gulf Biomedical Sciences Research Institute, Bushehr University of Medical Sciences, Bushehr 7514633341, Iran

**Keywords:** sono-photocatalyst, Cloisite 30B, wastewater, cationic dyes, scavenger

## Abstract

A new nanocomposite based on Cloisite 30B clay modified with ZnO and Ag_2_O nanoparticles (Cloisite 30B/ZnO/Ag_2_O) was synthesized as an effective catalyst in the sono-photocatalytic process of crystal violet (CV) and methylene blue (MB) dyes simultaneously. The characteristics and catalytic activity of Cloisite 30B/ZnO/Ag_2_O nanocomposite were investigated under different conditions. The specific active surface for Cloisite 30B/ZnO/Ag_2_O nanocomposite was 18.29 m^2^/g. Additionally, the catalytic activity showed that Cloisite 30B/ZnO/Ag_2_O nanocomposite (CV: 99.21%, MB: 98.43%) compared to Cloisite 30B/Ag_2_O (CV: 85.38%, MB: 83.62%) and Ag_2_O (CV: 68.21%, MB: 66.41%) has more catalytic activity. The catalytic activity of Cloisite 30B/ZnO/Ag_2_O using the sono-photocatalytic process had the maximum efficiency (CV: 99.21%, MB: 98.43%) at pH 8, time of 50 min, amount of 40 mM H_2_O_2_, catalyst dose of 0.5 g/L, and the concentration of ‘CV + MB’ of 5 mg/L. The catalyst can be reused in the sono-photocatalytic process for up to six steps. According to the results, •OH and h^+^ were effective in the degradation of the desired dyes using the desired method. Data followed the pseudo-first-order kinetic model. The method used in this research is an efficient and promising method to remove dyes from wastewater.

## 1. Introduction

Water is one of the important and necessary resources for the life and growth of plants and animals, and out of 71% of the water that covers the earth’s surface, only 3% of it is drinkable [1,2]. In recent years, the growth of industries, increase in urbanization, and other geological factors have caused the widespread pollution of water sources [3] and according to the surveys, every year, about 300–500 million tons of solvents, heavy metals, toxic sludge, and other industrial wastes are released into water sources [4]. One of the most common dangerous environmental pollutants is the pollutants caused by dyes in sewage, which are released into the environment as a result of various industrial activities, such as tanning, textile, paper making, paint, and pigment entering the environment [5,6]. The textile industry is one of the largest economic industries that consume a lot of dye and water every day, and according to the surveys, about 350 to 380 m^3^ water are used for each ton of textile goods, which constitutes 15–20% of industrial pollution [7] and contains 10–30% of the dye used in the textile industry [8,9]. The discharge of colored wastewater into the environment is one of the main concerns of human society for the environment because most dyes are harmful, carcinogenic, and teratogenic [10]. Dyes used in various industries are usually classified into cationic, anionic, and non-ionic groups based on the ionic charge carried by their structure. Cationic dyes have more toxicity than other ones [11]. Cationic dyes such as crystal violet (CV) and methylene blue (MB) can cause various diseases, such as breathing disorders, eye irritation, vomiting, increased heart rate, burning skin, cancer, shock and convulsions, cyanosis, mutagenicity, jaundice, blindness and eye irritation [12,13,14].

In recent years, various processes, such as biological purification, photocatalytic degradation, membrane separation, advanced oxidation processes, and surface adsorption, have been used to remove colored pollutants from aqueous solutions and the environment [15,16]. Among the mentioned methods, physical and physicochemical methods, although they have a good ability to clean the wastewater of industries containing dyes, they do not cause the decomposition of the pollutants in the wastewater and cause their transfer from one phase to another, in which case, they do not solve the environmental problem completely [17]. Therefore, it is necessary to use efficient and environmentally friendly methods to remove organic compounds, such as dyes, from the environment. For this purpose, the advanced oxidation process (AOP) is recommended as the best method to remove organic pollutants, such as dyes, from the aqueous solution, in which different techniques, such as photocatalysis, sonolysis, Fenton, used sono-photocatalysis, wet oxidation, and light-assisted Fenton [18]. In AOP processes, reactive oxygen species, such as hydrogen peroxide, hydroxyl radicals, peroxide radicals, and singlet oxygen, are produced, which can cause rapid oxidation of toxic organic pollutants [19,20] and turn them into non-hazardous or less toxic products, such as H_2_O, CO_2_ and mineral acids. Currently, all AOP methods are used to remove pollutants from aqueous solution, but these methods still have limitations such as requiring a long time and being chemically intensive or ineffective [21]. Among the AOP methods, the sono-photocatalytic process has received much attention in recent years, because this process is a combination of the sonolysis and photolysis methods in the presence of a semiconductor catalyst, which produces an integrated effect with a synergistic effect and causes rapid catalytic decomposition of organic pollutants [22]. The presence of solid particles (catalyst) in the sono-photocatalytic process enables the use of low ultrasonic power intensities and causes the formation of additional nucleation sites, which, as a result, increases the number of cavitation events and increases the performance of the degradation process [20].

In recent years, various compounds of metal oxides on the nanoscale, such as titanium oxide, zinc oxide, alumina, ceria, and silica, have been used in the process of organic pollutants degradation, among which titanium oxide and zinc oxide (ZnO), due to their strong oxidation property, non-toxicity, high photochemical corrosive resistance, and cost-effectiveness, have received much attention [23]. Due to its good thermal and chemical stability, low toxicity [24], high specific surface area, electrochemical activities, and stability in harsh process conditions [25], ZnO is one of the most widely used photocatalysts for the removal of organic pollutants, which has a band gap of 3.7 eV and high exciton-binding energy equal to 60 meV [26], and it is available in wide areas with low economic cost [27]. However, the commercialization of the organic pollutant degradation process based on ZnO has limitations because ZnO has a wide band gap, rapid recombination of valence band holes (h^+^)/conduction band electron(e^−^), low surface charge transfer rate, and difficulty in isolation and recovery [28]. Therefore, to overcome the mentioned limitations and improve the catalytic performance of ZnO, its doping and modification using metal oxides and other materials have been proposed. Another compound that has received much attention in photocatalytic processes in recent years is silver oxide (Ag_2_O). The results of the researchers have shown that this nanoparticle is efficient in the visible light region and combining it with a metal oxide with a wide band gap leads to an increase in its photocatalytic activity [29,30]. Ag_2_O is a p-type semiconductor that has a band gap of 1.2 eV and is widely used in water and catalyst purification [31,32,33]. Silver nanoparticles have different functions, such as (1) improving light absorption due to the surface plasmon effect, (2) serving as electron sinks and promoting surface electron transfer, and (3) increasing photocatalytic performance due to charge carrier lifetime, which makes them widely has been used [34,35]. However, recovering silver nanoparticles from aqueous solutions and reusing them is a difficult task. To solve this problem, silver nanoparticles must be placed on inert and motionless supports to prevent the accumulation of their particles and increase their stability to facilitate their finding and separation [36].

In this study, Ag_2_O nanoparticles were first placed on Cloisite 30B nanoclay, and then the surface of this nanocomposite was modified using ZnO nanoparticles. After the synthesis of the photocatalyst, its properties were investigated using various techniques, such as XRD, BET, SEM, Map-EDX, XPS, and FTIR. To investigate the role of ZnO and Ag_2_O nanoparticles and the interaction of their synergistic effect on the efficiency of the simultaneous degradation process of CV and MB dyes, the sono-photocatalytic process was studied discontinuously in the presence of UV light and ultrasonic waves, and the effect of parameters such as pH, reaction time, catalyst dosage, the amount of H_2_O_2_, the initial concentration of the desired dyes and the effect of common ions in the aqueous solution was investigated. After performing the sono-photocatalytic process, the kinetic study and the reusability of the desired catalyst were studied for up to 6 steps, and the active radical species were evaluated for simultaneous degradation of CV and MB dyes.

## 2. Materials and Methods

### 2.1. Materials

Crystal violet (C_25_H_30_N_3_Cl, MV: 407.98 g/mol, purity: >99.9, and λ_max_: 590 nm) and methylene blue (C_16_H_18_ClN_3_S, MV: 319.86 g/mol, purity: >99.9, and λ_max_: 664 nm) dyes, hydrogen peroxide (H_2_O_2_, 30%), hydrochloric acid (HCl, 37%), sodium hydroxide (NaOH), zinc chloride (ZnCl_2_), isopropyl alcohol (IPA), ethylene diamine tetra acetic acid disodium (EDTA-2Na), benzoquinone (BQ), silver nitrate (AgNO_3_), and sodium dodecyl sulfate (NaC_12_H_25_SO_4_) in laboratory grade were prepared and used from Merck (Kenilworth, NJ, USA) and Aldrich companies (St. Louis, MO, USA). Cloisite 30B nanoclay was purchased from Southern Clay Products Inc. (Gonzales, TX, USA) and was used as a support in the synthesis of the desired photocatalyst.

### 2.2. Preparation of Cloisite 30B/ZnO/Ag_2_O Photocatalyst

To prepare the desired photocatalyst, first, a Zn^2+^ solution with a concentration of 1 M was prepared and 0.7 g of Cloisite 30B nanoclay was added to it and stirred for 50 min using a magnetic stirrer. After that, NaOH solution with a concentration of 2 M was added dropwise and it was stirred at room temperature for 3 h using a magnetic stirrer. Cloisite 30B/ZnO suspension was separated and after washing and drying, it was placed in a thermal furnace at a temperature of 500 °C for 4 h at a temperature rate of 5 °C/min, and then it was powdered and used as a photocatalyst.

**Figure 1:**
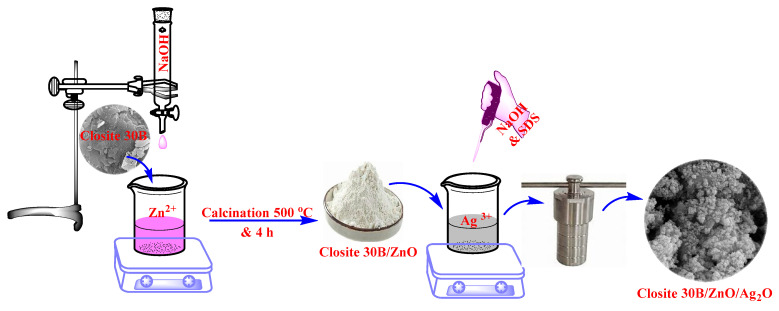
Schematic view of Cloisite 30B/ZnO/Ag_2_O photocatalyst synthesis process.

In the next step, the hydrothermal method was used for the synthesis of Cloisite 30B/ZnO/Ag_2_O. For this purpose, first, 40 mL of AgNO_3_ solution with a concentration of 0.5 M was prepared and 1 g of prepared Cloisite 30B/ZnO was added to it and stirred for 30 min using a magnetic stirrer and then transferred to a Teflon-lined chamber with a volume 100 mL. After that, 40 mL of sodium dodecyl sulfate (SDS) solution with a concentration of 0.1 M and 20 mL of NaOH solution with a concentration of 1 M were added to the desired mixture and placed at 120 °C for 12 h. After the mentioned time, the desired nanocomposite was separated from the aqueous solution and the desired sample was washed using distilled water and ethanol and placed at 100 °C for 24 h to dry completely. For the synthesis of the Cloisite 30B/Ag_2_O sample, the same method was used, with the difference that pure Cloisite 30B nanoclay was used. The characteristics of Cloisite 30B/ZnO/Ag_2_O nanocomposite were performed based on the techniques presented in previous studies [37]. Figure 1 shows the schematic of the synthesis process of Cloisite 30B/ZnO/A_g2_O photocatalyst.

### 2.3. Assessment of Sono-Photocatalytic Activity

The photocatalytic activity of Cloisite 30B/ZnO/Ag_2_O nanocomposite in the simultaneous removal of MB and CV dyes was carried out discontinuously in a steel tube reactor, in the center of which a glass tube is embedded to place the UV (254 nm) light source. The sono-photocatalytic activity of Cloisite 30B/ZnO/Ag_2_O nanocomposite was performed in the presence of ultrasonic waves (24 kHz, 180 W) and a UV lamp with 15 W power. In this study, the effect of parameters, such as reaction time (5–60 min), H_2_O_2_ amount (10–90 mM), catalyst amount (0.1–0.9 g/L), pH (2–9), initial concentration of desired dyes (5–25 mg/L) and the effect of different anions (CO_3_^2−^, Cl^−^, NO_3_^−^ and PO_4_^3−^) on the catalytic activity of Cloisite 30B/ZnO/Ag_2_O nanocomposite was studied. Normally, to study the effect of the mentioned parameters on the catalytic activity, first, 100 mL of an aqueous solution containing a certain concentration of the desired dyes was prepared and its pH was adjusted using NaOH and HCl solution with a concentration of 0.1–1 M in the desired pH range. Then, a specific amount of Cloisite 30B/ZnO/Ag_2_O nanocomposite was added to the aqueous solution and mixed in a dark environment for 30 min using a magnetic stirrer until the adsorption–desorption balance between the solid phase and the aqueous solution before UV light and ultrasonic waves to be established. After establishing the adsorption–desorption balance, a certain amount of H_2_O_2_ (in the desired range) was added to the aqueous solution, and the desired mixture was added to the tubular reactor, placed in the ultrasonic bath at ambient temperature and was investigated in the time range of 5–60 min. After the intended process, the Cloisite 30B/ZnO/Ag_2_O nanocomposite was separated from the aqueous solution by centrifugation (5 min, 4000 rpm) and the concentration of MB and CV dyes remaining in the aqueous solution was analyzed using a spectrophotometer equipped with UV-Vis at 665 and 592 nm wavelengths, respectively. The removal percentage of the desired dyes was determined using Cloisite 30B/ZnO/Ag_2_O nanocomposite photocatalyst from Equation (1):

(1) $$\mathrm{Removal}\;(\%)=\frac{{\mathrm{dye}}_{i}-{\;\mathrm{dye}}_{e}}{{\mathrm{dye}}_{i}}\times 100$$

where dye_i_ and dye_e_ (mg/L) are the initial and equilibrium concentrations of the desired dyes, respectively.

After investigation and determining the optimal parameters effective in removing the desired dyes using Cloisite 30B/ZnO/Ag_2_O nanocomposite, the scavenger effect, the ability to regenerate and reuse, as well as the ability of the desired catalyst to the treatment of the industrial wastewater in the optimal conditions with the determined parameters were investigated.

## 3. Results and Discussion

### 3.1. Properties of the Photocatalyst

**Figure 2:**
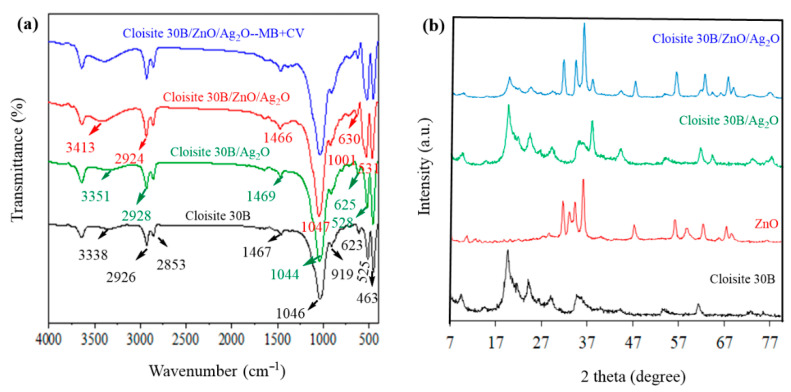
(**a**) FTIR analysis for Cloisite 30B, Cloisite 30B/Ag_2_O, and Cloisite 30B/ZnO/Ag_2_O samples before and after the simultaneous catalytic removal process of dyes and (**b**) XRD analysis for Cloisite 30B, ZnO, Cloisite 30B/Ag_2_O and Cloisite 30B/ZnO/Ag_2_O.

FTIR spectra for Cloisite 30B, Cloisite 30B/Ag_2_O, and Cloisite 30B/ZnO/Ag_2_O samples before and after the catalytic removal process of CV and MB dyes are shown in Figure 2a. Based on the FTIR results, a high-intensity absorption peak was observed in the structure of Cloisite 30B in the wavenumber range of 3338 cm^−1^, which after modification of Cloisite 30B using Ag_2_O and Ag_2_O/ZnO changed in the range of 3351 and 3413 cm^−1^ respectively, which can be caused by the vibrations of the stretching group –OH (water molecule) in the structure of the mentioned samples [38]. In addition, in the structure of Cloisite 30B in the ranges of 1647, 1467, 1046, 919, 725, 623, 524, and 463 cm^−1^ vibrations appeared that can be caused by H-O-H, C-O-C, Si-O, Al-OH, Al/Mg-OH, Al-OH, Si-O, and Si-O vibrations, respectively [39]. After the modification of Cloisite 30B using Ag_2_O and Ag_2_O/ZnO, there were changes in the intensity and position of absorption peaks in the structure of Cloisite 30B, which can be due to the interaction between functional groups and metal oxides of Ag_2_O and Ag_2_O/ZnO [35,40]. After the catalytic degradation process of CV + MB dyes, no significant changes were made in the structure of the catalyst, which indicates that the desired catalyst is stable in the sono-photocatalytic process and the side products formed due to the dye compounds are not placed on its surface.

In Figure 2b, XRD spectra for Cloisite 30B, ZnO, Cloisite 30B/Ag_2_O and Cloisite 30B/ZnO/Ag_2_O samples are presented. In the structure of Cloisite 30B, a peak is observed in the range of 2θ equal to 7.34^o^, which corresponds to the interlayer distance of 7.6 Å, where, after modification of Cloisite 30B using Ag_2_O and Ag_2_O/ZnO, the range of this peak and the distance between the layers increased. The increase in the interlayer distance after modification shows that the layers in the Cloisite 30B structure are well exfoliated [41] and the desired metal oxides are well placed in the Cloisite 30B layers. In addition, in the structure of Cloisite 30B, peaks with different intensities appeared in the range of 17–80°, and these peaks can be caused by the quartz present in the sample structure and show that Cloisite 30B has a crystalline structure [42]. In the structure of ZnO in the range of 7–80°, peaks with different intensity and sizes have appeared, which indicate crystal planes (100), (002), (101), (102), (110), (103) and (112) that exist in the ZnO structure [43]. After modification of Cloisite 30B using Ag_2_O nanoparticles, peaks in the range of 37.39, 55.37, 64.34, and 77.29° appeared, indicating the presence of Ag_2_O nanoparticles in the structure of Cloisite 30B [44,45]. After modification of Cloisite 30B using oxides of Ag_2_O and ZnO nanoparticles, the peaks related to the crystalline phases of Cloisite 30B, Ag_2_O and ZnO completely appeared next to each other, which shows that the constituent components of the nanocomposite have a suitable interaction and the desired nanocomposite has been successfully synthesized.

**Figure 3:**
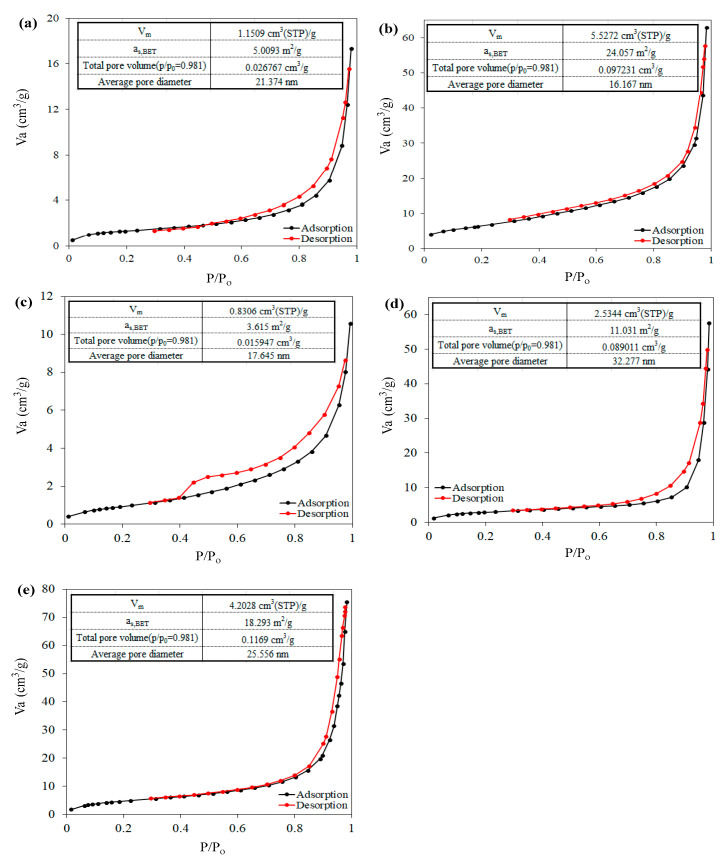
N_2_ adsorption–desorption isotherm at temperature of 77 K for (**a**) Cloisite 30B, (**b**) ZnO, (**c**) Ag_2_O, (**d**) Cloisite 30B/Ag_2_O and (**e**) Cloisite 30B/ZnO/Ag_2_O samples.

BET analysis was used to investigate the specific active surface and also the effect of the constituents of Cloisite 30B/ZnO/Ag_2_O nanocomposite on the specific active surface and pore volume (Figure 3). Based on the mentioned results, the value of specific active surface for Cloisite 30B, ZnO and Ag_2_O samples was determined to be 5.0093, 24.057, and 3.615 m^2^/g, respectively. After modification of Cloisite 30B using Ag_2_O and ZnO/Ag_2_O metal oxides, the specific active surface of Cloisite 30B clay increased to 11.031 and 18.293 m^2^/g, respectively. The increase in the specific active surface of the mentioned clay with the use of the desired metal oxides can be caused by the formation of different oxide structures [46] and the placement of metal oxides between the layers of the clay; as a result, the pores increase. These results are in good agreement with the presented XRD analysis (Figure 2b). In addition, the pore volume in the Cloisite 30B structure was determined to be 0.026 cm^3^/g, and when it was modified using Ag_2_O and ZnO/Ag_2_O metal oxides, the pore volume increased to 0.089 and 0.116 cm^3^/g, respectively, such that it can confirm the increase in the distance between clay layers and the placement of metal oxides in its structure. In addition, it should be mentioned that the hysteresis loop for all the presented samples is of type IV and the mentioned samples are classified in the category of mesoporous materials based on IUPAC standards.

**Figure 4:**
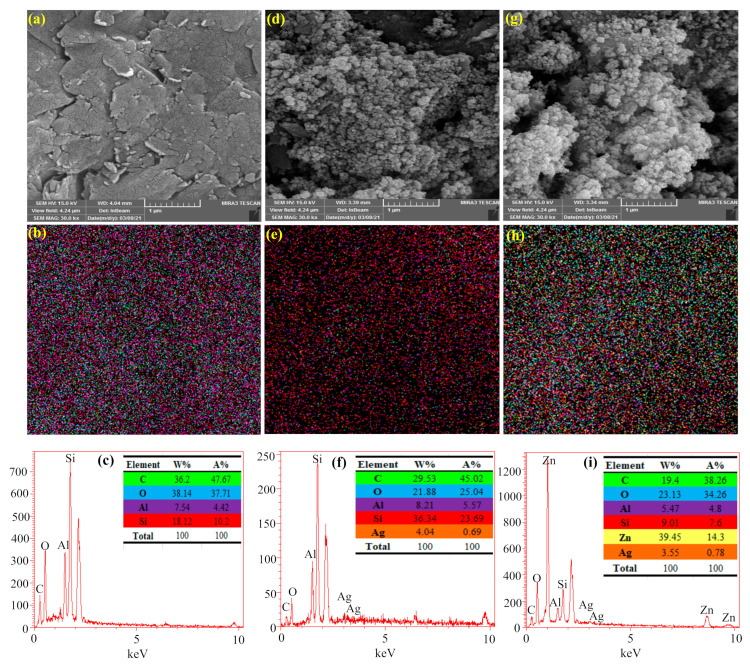
SEM and Map-EDX analysis for (**a**–**c**) Cloisite 30B, (**d**–**f**) ZnO, and (**g**–**i**) Cloisite 30B/ZnO/Ag_2_O.

SEM and Map-EDX analyses were used to investigate the morphology and surface changes in the Cloisite 30B sample before and after modification using Ag_2_O and ZnO/Ag_2_O metal oxides, and the results are presented in Figure 4. As shown in Figure 4a, Cloisite 30B clay has a layered structure with different pore sizes in its structure, with elements C (36.2 wt.%), O (38.14 wt.%), Al (7.54 wt.%), and Si (18.12 wt.%), and these elements have a uniform distribution throughout the sample (Figure 4b,c). After modification of Cloisite 30B using Ag_2_O and ZnO/Ag_2_O metal oxides, particles with spherical morphology and different sizes appeared on the surface of Cloisite 30B, which can confirm the existence of the mentioned metal oxides (Figure 4d,g). Map-EDX analysis was also used to confirm the presence of metal oxides in the Cloisite 30B structure, and the results showed that there are Zn and Ag elements in the Cloisite 30B structure after modification (Figure 4e,f,h,i). Therefore, based on the obtained results, it can be claimed that Cloisite 30B clay is successfully modified using the desired metal oxides and has a suitable interaction.

**Figure 5:**
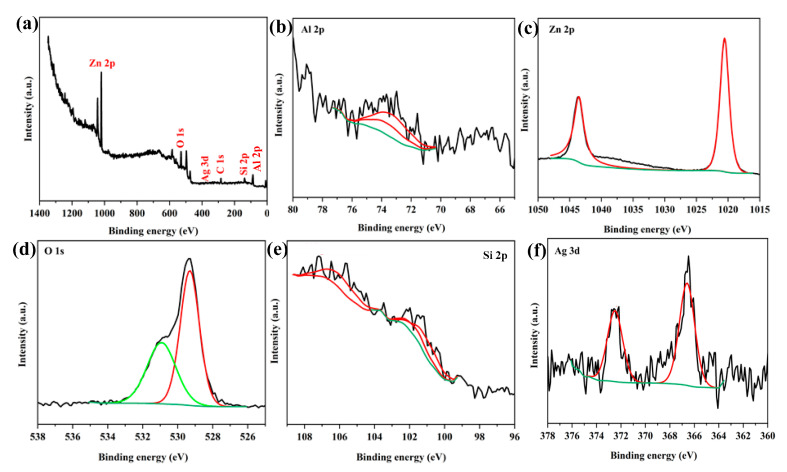
Spectra from XPS analysis for (**a**) Cloisite 30B/ZnO/Ag_2_O sample, and regional high-resolution XPS spectra of (**b**) Al 2p, (**c**) Zn 2p, (**d**) O 1s, (**e**) Si 2p, and (**f**) Ag 3d.

The results of X-ray photoelectron spectrometry (XPS) analysis for the Cloisite 30B/ZnO/Ag_2_O nanocomposite are presented in Figure 5. The full scan spectrum related to XPS analysis showed that in the structure of the Cloisite 30B/ZnO/Ag_2_O nanocomposite, different peaks are related to C, Ag, O, Al, Si, and Zn elements (Figure 5a). For the energy region related to Al 2p, in the range of 73.75 eV, peaks derived from Al_2_O_3_ were observed [47] and are caused by the presence of Cloisite 30B clay in the Cloisite 30B/ZnO/Ag_2_O nanocomposite structure (Figure 5b). In the Cloisite 30B/ZnO/Ag_2_O photocatalyst structure, a high-resolution spectrum appeared in the range of 1010–1050 eV (Figure 5c), which has two peaks at 1020.88 and 1044.3 eV, respectively, indicating that the electronic states of Zn 2p_3/2_ and Zn 2p_1/2_ are related to Zn^2+^ [47,48], confirming that ZnO nanoparticles are included in the catalyst structure. In Figure 5d, the O 1s signal shows that it has two peaks in the range of binding energies of 529.5 and 531.18 eV, which indicates the existence of two different types of oxygen (O) in the structure of Cloisite 30B/ZnO/Ag_2_O nanocomposite. Based on previous studies, the binding energies of 529.5 and 531.18 eV in the O 1s spectrum are related to O^2−^ ions surrounded by Ag (Ag_2_O) and Zn (ZnO) ions, respectively [49], which show that ZnO and Ag_2_O nanoparticles are successfully placed in the structure of the Cloisite 30B/ZnO/Ag_2_O nanocomposite and have a good interaction with each other. Additionally, the spectrum related to Si 2p in the binding energy range of 102.18 eV has a symmetrical peak that is related to the Si-O bond (Figure 5e) and shows that there is a uniform bond [50]. Additionally, the binding energies of 366.68 and 372.48 eV are attributed to Ag 3d_5/2_ and Ag 3d_3/2_ (Figure 5f), which shows that in the Cloisite 30B/ZnO/Ag_2_O photocatalyst structure, the Ag^+^ ion in Ag_2_O exists [51], and it is consistent with the results of the XRD and EDX analysis.

### 3.2. The Effect of pH and the Role of the Catalyst

**Figure 6:**
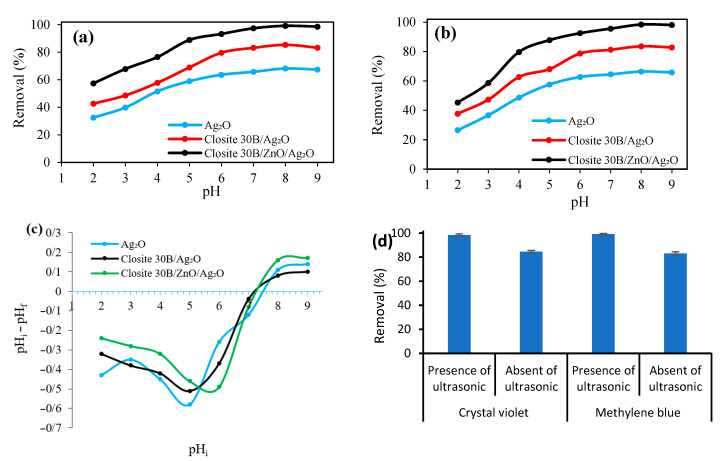
The pH effect on the removal efficiency of (**a**) crystal violet and (**b**) methylene blue using Ag_2_O, Cloisite 30B/Ag_2_O and Cloisite 30B/ZnO/Ag_2_O (dyes concentration: 5 mg/L, reaction time: 50 min, catalyst dosage: 0.5 g/L, amount of H_2_O_2_: 40 µL/100 mL) and (**c**) pH_i_-pH_f_ versus pH_i_ to determine pH_zpc_, (**d**) the efficiency of dye removal in the presence and absence of ultrasonic waves.

The initial pH is one of the effective parameters in photocatalytic processes because the activity of the photocatalytic process is influenced by the surface charge of the material, the molecular charge, the adsorption of organic pollutants on the surface of the photocatalyst, and the number of hydroxyl radicals in the aqueous solution [52]. For this purpose, in this study, we changed the initial pH in the range of 2–9 and investigated its effect on the simultaneous removal process of CV and MB cationic dyes using Ag_2_O, Cloisite 30B/Ag_2_O, and Cloisite 30B/ZnO/Ag_2_O samples and the results are presented in Figure 6a,b. As the results have shown, the maximum efficiency of the simultaneous degradation of MB and CV dyes using Ag_2_O, Cloisite 30B/Ag_2_O, and Cloisite 30B/ZnO/Ag_2_O samples took place at the initial pH = 8. At pH values lower and higher than 8, the efficiency of the process decreased, which can be interpreted according to the number of zero charges (pH_zpc_) of Ag_2_O, Cloisite 30B/Ag_2_O and Cloisite 30B/ZnO/Ag_2_O samples. Based on the presented results (Figure 6c), the values of pH_zpc_ for Ag_2_O, Cloisite 30B/Ag_2_O, and Cloisite 30B/ZnO/Ag_2_O samples were determined to be 7.5, 7.2, and 7.32, respectively, which shows that the surface of the desired samples at pH > pH_zpc_ and at pH < pH_zpc_ has negative and positive surface charges due to the adsorption of hydroxide anions and the adsorption of protons on its surface, respectively. At pH < pH_zpc_, the surface of the desired catalyst samples has a positive charge, and since CV and MB dyes also have positive charges, an electrostatic force of repulsion is created between them, which prevents dye molecules from approaching the surface of the catalysts [53]. Additionally, at pH > pH_zpc_, the concentration of ions and hydroxyl radicals increase, and the aforementioned radicals can be converted into oxide ions (O^−^) [54]; as a result, it causes a decrease in the concentration of hydroxide radicals and a decrease in the efficiency of the dyes degradation process. In addition, the results showed that the order of photocatalytic degradation activity of the samples is Ag_2_O < Cloisite 30B/Ag_2_O < Cloisite 30B/ZnO/Ag_2_O, which can be caused by the specific active surface and higher pore volume that can be effective in the catalytic degradation process [55]. Therefore, it can be mentioned that the catalytic degradation property of the Cloisite 30B/ZnO/Ag_2_O nanocomposite increased significantly due to the synergistic coupling of ZnO, Ag_2_O, and Cloisite 30B. In the following, to investigate the simultaneous degradation of CV and MB dyes, Cloisite 30B/ZnO/Ag_2_O nanocomposite was used as a photocatalyst in the sono-photocatalytic degradation process.

Figure 6d also explores the efficiency of dye removal in the presence and absence of ultrasonic waves. In this figure, it is clear that ultrasonic waves contributed about 13% and 16% efficiency in the removal efficiency of crystal violet and methylene blue dyes. Ultrasonic waves can remove pollutants by creating hydroxyl radicals. The role of ultrasonic waves was proven in other research [56,57].

### 3.3. The Effect of H_2_O_2_ Content, Amount of Photocatalyst, Duration of Reaction, and Dyes Concentration

**Figure 7:**
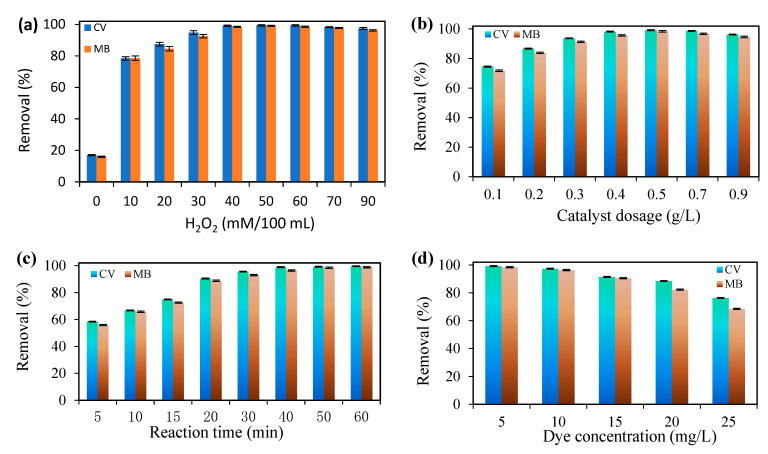
(**a**) Effect of amount of H_2_O_2_ (concentration of dyes 5 mg/L, reaction time 50 min, catalyst dose 0.5 g/L and pH = 8), (**b**) Effect of catalyst dose (initial concentration of dyes 5 mg/L, reaction time 50 min, amount of H_2_O_2_ 40 mM/100 mL and pH = 8), (**c**) effect of reaction time (initial concentration of dyes 5 mg/L, dose of catalyst 0.5 g/L, amount of H_2_O_2_ 40 mM/100 mL and pH = 8), and (**d**) the effect of the dyes concentration (catalyst dose 0.5 g/L, reaction time 50 min, amount of H_2_O_2_ 40 mM/100 mL and pH = 8) on the dye removal using Cloisite 30B/ZnO/Ag_2_O.

The amount of H_2_O_2_ is one of the effective factors in advanced oxidation processes because H_2_O_2_ can affect the number of free radicals in the reaction environment. In this study, for the simultaneous degradation of CV and MB dyes using Cloisite 30B/ZnO/Ag_2_O photocatalyst, the amount of H_2_O_2_ in the range of 10–90 mM was investigated, and the results are shown in Figure 7a. The obtained experimental results showed that by increasing the amount of H_2_O_2_ from 0 to 40 mM, the efficiency of the simultaneous degradation process of CV and MB dyes increased from 17 and 16% to 99.21 and 98.43%, respectively, and this increase in efficiency can be due to the increase in hydroxyl radicals produced by using H_2_O_2_ in the reaction medium. After the amount of 40 mM of H_2_O_2_, the efficiency of the process did not change significantly, and in the amount of 70–90 mM of H_2_O_2_, the efficiency of the degradation process of the desired dyes decreased, which could be due to the inhibition of hydroxyl radicals produced by H_2_O_2_ which has also been reported in previous studies [58]. When the amount of hydrogen peroxide was zero, partial removal efficiency (about 15%) for dyes occurred, which was caused by UV rays or the absorption of dyes into the catalyst. To better understand the radicals produced by H_2_O_2_ in the presence of UV light and ultrasonic waves, as well as the inactivation of hydroxyl radicals using H_2_O_2_, the relevant possible mechanisms are presented below:

(2) $$H_{2}O_{2}+\mathrm{Ultarsonic}\;/\;\mathrm{hv}\to 2^{•}OH$$

(3) $$H_{2}O_{2}{+}^{•}\mathrm{OH}\to H^{•}O_{2}+{\;H}_{2}O$$

(4) $${}^{•}\mathrm{OH}+{}^{•}OH\to H_{2}O_{2}$$

(5) $${}^{•}{\mathrm{HO}}_{2}+H_{2}O_{2}\to^{•}\mathrm{OH}+H_{2}O+O_{2}$$

(6) $${}^{•}{\mathrm{HO}}_{2}{+}^{•}{\mathrm{HO}}_{2}\to H_{2}O_{2}+O_{2}$$

(7) $${}^{•}{\mathrm{HO}}_{2}{+}^{•}\mathrm{OH}\to H_{2}O+O_{2}$$

According to Equation (2), H_2_O_2_ in the presence of ultrasonic waves or UV light turns into hydroxyl radicals and reacts with dye molecules and causing their degradation. When the amount of H_2_O_2_ used increases, the number of hydroxyl radicals produced in the reaction medium increases, and they are deactivated due to their collision with H_2_O_2_ in the aqueous solution (Equation (3)). In addition, •OH radicals in high concentrations can collide with each other and dimer with each other and become H_2_O_2_ (Equation (4)). In Equations (5)–(7), other possible interactions between •OH and other molecules are also presented, which in turn can cause the reduction in •OH radicals in the aqueous solution and as a result, reduce the efficiency of the dye degradation process [59].

The catalyst dosage parameter is another important and vital parameter in advanced oxidation processes that can increase or decrease process efficiency [19]. Therefore, in the present study, the effect of the catalyst dose is in the range of 0.1–0.9 g/L with laboratory conditions such as pH = 8, reaction time 50 min, amount of H_2_O_2_ equal to 40 mM, dye concentration 5 mg/L, and the presence of ultrasonic waves (24 kHz, 180 W); the results are presented in Figure 7b. The efficiency of the simultaneous degradation of CV and MB dyes using the sono-photocatalytic process increased from 74.54 and 71.73 to 99.21 and 98.43%, respectively, by increasing the catalyst dose from 0.1 to 0.5 g/L; after the amount of 0.5 g/L from the catalyst, the efficiency of the degradation process of the desired dyes decreased. The increase in the degradation efficiency of the desired dyes by increasing the amount of catalyst can be due to various factors, such as the production of hydroxyl radicals, superoxide radicals [60], more adsorption of dye molecules in the active sites of the catalyst [61], and improved cavitational activity [62]. In addition, it should be mentioned that the decrease in the degradation efficiency of the desired dyes after the optimal amount of catalyst (0.5 g/L) can also be attributed to various factors, such as the accumulation of catalyst particles, the formation of a suspension in the aqueous solution by the catalyst, and the reduction in light penetration into the aqueous solution [63].

In Figure 7c, the effect of reaction time on the efficiency of the simultaneous degradation process of CV and MB dyes using the sono-photocatalyst process is investigated as an effective parameter in the time range of 5–60 min. Based on the obtained results, the efficiency of the simultaneous destruction process of CV and MB dyes is low in the initial times, and with the passage of reaction time, the efficiency of the dye destruction process was gradually improved. The low efficiency of the sono-photocatalyst process during the initial reaction time can be caused by the formation of insufficient amounts of free radicals that cannot completely degrade the dye molecules in the aqueous solution [64]. When the reaction time increases, the number of free radicals in the reaction environment increases, sufficient time is provided for the interaction between free radicals and dye molecules, and more dye molecules are degraded.

Another parameter that can affect the efficiency of the degradation process of the desired dyes is the concentration of the dye pollutant in the aqueous solution. For this purpose, to investigate the effect of the initial concentration of CV and MB dyes on the simultaneous degradation efficiency of the desired dyes using the sono-photocatalytic process, the concentration of dyes in the range of 5–25 mg/L was investigated, and the results are presented in Figure 7d. As the results show, by increasing the initial concentration of CV and MB dyes from 5 to 25 mg/L, the efficiency of the degradation process decreased from 99.21 and 98.43% to 76.32 and 68.54%, respectively. The decrease in the efficiency of the degradation process with the increase in the initial concentration of the desired dyes can be attributed to various factors, such as the limitation of light penetration into the dye solution, the reduction of heat energy emission produced by ultrasonic cavitation, the increase in the electrostatic force of repulsion between the dyes adsorbed on the surface and the photocatalyst layers with dye molecules in the aqueous solution [65], which is attributed the coating of the catalyst surface and the active sites in its structure using dye molecules, which resulted in a decrease in the production of OH• and •O^2−^ radicals. The efficiency of the process of degradation dyes is effective [66].

### 3.4. The Investigation of Common Anions Effect, Scavengers Effect, and Photo-Catalyst Reuse

**Figure 8:**
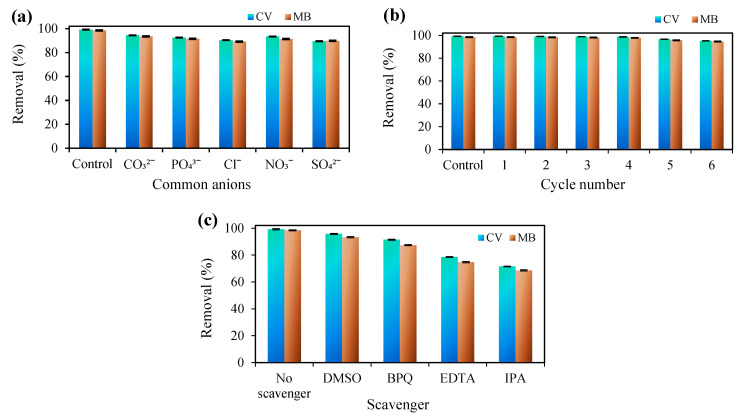
(**a**) The effect of common anions on the efficiency of the dye degradation using the sono-photocatalytic process, (**b**) the ability to reuse the Cloisite 30B/ZnO/Ag_2_O catalyst in the degradation of dyes and (**c**) the effect of different scavengers on the efficiency of the dyes degradation (dye concentration: 5 mg/L, catalyst dose: 0.5 g/L, reaction time: 50 min, amount of H_2_O_2_: 40 mM/100 mL, and pH = 8).

Today, one of the issues that has received attention in the field of the environment today is the use of catalysts and adsorbents in industrial wastewater treatment. Industrial wastewater usually contains inorganic salts along with the pollutant, which can affect the efficiency of the pollutant removal process. For this purpose, the effect of CO_3_^2−^, PO_4_^3−^, Cl^−^, NO_3_^−^ and SO_4_^2−^ anions at 5 mM concentrations on the efficiency of the simultaneous degradation process of MB and CV dyes with a concentration of 5 mg/L was investigated, and the results are shown in Figure 8a. According to the determined results, the presence of all anions in the aqueous solution reduced the efficiency of the sono-photocatalytic process in the simultaneous degradation of CV and MB dyes and prevented the degradation of the desired dyes. After performing the sono-photocatalytic process in laboratory conditions, duration of 50 min, photocatalyst dose 0.5 g/L, initial concentration of desired dyes 5 mg/L, pH = 8 and H_2_O_2_ 40 mM, the efficiency of the degradation process of CV and MB dyes was 99.21 and 98.43% in the presence of CO_3_^2−^, PO_4_^3−^, Cl^−^, NO_3_^−^ and SO_4_^2−^ anions decreased to 94.43 and 93.52%, 92.54 and 91.42%, 90.37 and 89.23%, 93.44 and 91.32% and 89.52 and 89.78, respectively. The decrease in the efficiency of the sono-photocatalytic process in the presence of anions can be caused by hole scavenging and the trapping of OH• and •O^2−^ radicals by the desired anions and the production of less efficient radicals [67,68].

To further confirm the ability of Cloisite 30B/ZnO/Ag_2_O as an effective photocatalyst in the simultaneous degradation process of dyes from aqueous solution using the sono-photocatalytic process, the recycling and reuse of Cloisite 30B/ZnO/Ag_2_O photocatalyst were investigated in the degradation of the desired dyes. The reusability of Cloisite 30B/ZnO/Ag_2_O photocatalyst was investigated in laboratory conditions for 50 min, photocatalyst dose 0.5 g/L, initial concentration of desired dyes 5 mg/L, pH = 8 and H_2_O_2_ 40 mM, and the obtained results are presented in Figure 8b. The reusability of the desired photocatalyst was investigated up to 6 stages, and for each stage, the photocatalyst was separated from the aqueous solution and after washing several times with deionized water, it was placed at a temperature of 90 °C for 120 min until completely to dry. The investigation of the reuse of the desired photocatalyst showed that no significant changes in the efficiency and activity of the catalyst were observed in up to 4 stages. After 4 stages of reuse, the activity of the catalyst decreased, and in this case, in 6 stages of reuse, the efficiency of the sono-photocatalytic process in the simultaneous degradation of CV and MB dyes was determined to be 95.17 and 94.54%, respectively. The obtained results showed that the Cloisite 30B/ZnO/Ag_2_O nanocomposite has a high ability to simultaneously degrade dyes from an aqueous solution and can be easily recycled and reused in the sono-photocatalytic process.

In advanced oxidation processes, reactive species, such as anionic oxygen radicals, hydroxyl radicals, holes, and electrons, are produced using light and ultrasonic waves, which are effective in the oxidation of dye molecules [69]. To investigate the role of active species in the simultaneous degradation of CV and MB dyes, the sono-photocatalytic process in optimal conditions and the presence of benzoquinone (BQ, •O^2−^ scavenger), isopropanol (IPA, •OH), dimethyl sulfoxide (DMSO, electron scavenger) and ethylene Diamine tetra-acetic acid (EDTA, hole (h^+^) scavenger) was used as an active species adsorbent, and the results are shown in Figure 8c. As can be seen, the sono-photocatalytic activity of Cloisite 30B/ZnO/Ag_2_O has the highest efficiency in the absence of the desired scavengers, but in the presence of scavengers, the efficiency of the process decreases. On the other hand, the efficiency of the sono-photocatalytic process decreases significantly with the addition of EDTA and IPA scavengers, compared to other scavengers used. The decrease in the efficiency of dyes degradation with the addition of IPA and EDTA scavengers shows that the reactive species OH• and h^+^ have an essential role in the simultaneous degradation of CV and MB dyes, and the reactive species OH• has a more effective role than h^+^ in the sono-photocatalytic process.

### 3.5. Evaluation of the Toxicity of the Treated Aqueous Solution

**Figure 9:**
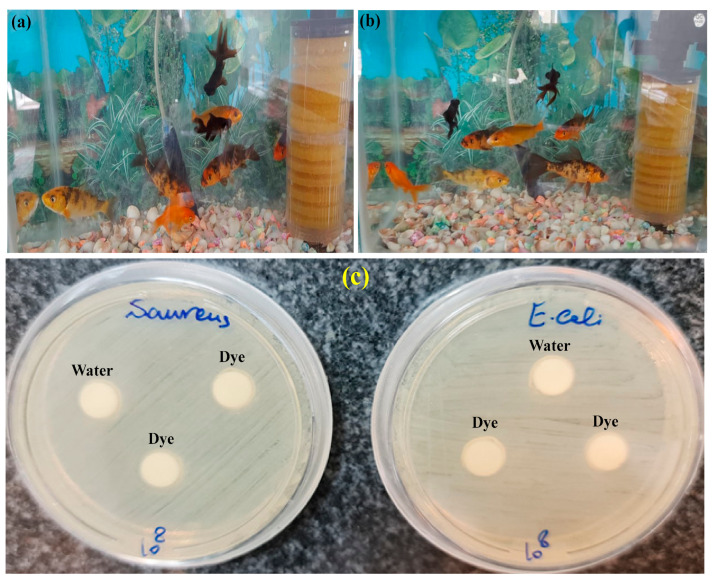
Examining the effect of toxicity of aqueous solution containing by-products of degraded dyes on aquarium fish (**a**) duration of 7 days and (**b**) duration of 20 days, (**c**) effect of toxicity of treated aqueous solution on microorganisms *S. aureus* and *E. coli*.

During the sono-photocatalytic process, as a result of the oxidation of organic pollutants such as dyes, by-products may be produced that are more dangerous than the main pollutant and are harmful to microorganisms and other living organisms in the water. To further investigate the by-products produced due to the simultaneous degradation of MB and CV dyes, the aqueous solution was subjected to the sono-photocatalytic process for 50 min at pH = 8, and after the separation of the catalyst, it was evaluated using GC-Mass analysis. The results of GC-Mass analysis showed that MB and CV dye molecules were converted into different compounds during the sono-photocatalytic process, but their amounts are less than 0.05 µg (Table 1). After treatment, industrial wastewater is usually released into the environment, and if the by-products produced are caused by toxic organic compounds, they can endanger the lives of microorganisms and other living organisms. To use the aqueous solution used in agriculture and aquaculture, the toxicity effect of the aqueous solution containing MB and CV dyes after the sono-photocatalytic process was investigated on aquarium fish. For this purpose, in an aquarium with a volume of 35 L, first, 15 L of treated water solution was mixed with 15 L of water and added to the aquarium, and eight fish of different sizes were added to it and observed for 20 days. It should be noted that the food program of the mentioned fish was performed every 8 h and during the study, the system was continuously aerated. After 20 days, all the fish placed in the aquarium were alive and no death was observed among them (Figure 9a,b).

In addition to investigating the toxicity effect of the treated aqueous solution on aquarium fish, the toxicity of the treated aqueous solution for microorganisms was also investigated after the sono-photocatalytic process in the presence of Cloisite 30B/ZnO/Ag_2_O nanocomposite. For this purpose, the effect of the toxicity of aqueous solution on *S. aureus* and *E. coli* microorganisms was studied using the disk diffusion method. First, the disk plates were placed in the treated aqueous solutions for 48 h to saturate the disk plates using the aqueous solution. Additionally, the desired microorganisms were cultured in the autoclaved nutrient broth culture medium and placed in an incubator at 37 °C for 24 h. After cultivating the desired microorganisms, the discs saturated with the treated aqueous solution were transferred to the Mueller Hinton agar culture medium, and then the bacterial solution with a concentration of 10^8^ was prepared with McFarland’s half and added to the Mueller Hinton agar culture medium, then it was placed in an incubator at 37 °C for 18 h. After the mentioned time, no bacterial growth was observed in the bacterial culture plates, which confirms that the aqueous solution after the sono-photocatalytic process is not dangerous for microorganisms (Figure 9c).

### 3.6. Kinetic Study and Comparison of Photocatalyst Performance

To investigate the kinetic behavior of the Cloisite 30B/ZnO/Ag_2_O sono-photocatalytic process in the simultaneous removal of CV and MB cationic dyes from aqueous solution, pseudo-first-order (PFO) and pseudo-second-order (PSO) kinetic models were used (Equations (8) and (9)):

(8) $${\ln\;(C}_{0}{/C)=k}_{1}.t$$

(9) $$\frac{1}{C}=\frac{1}{C_{0}}{+k}_{2}.t$$

Here, C_0_ (mg/L) is the concentration of cationic dyes before the start of the sono-photocatalytic degradation process, and C (mg/L) is the concentration of cationic dyes after the start of the sono-photocatalytic degradation process at times t (min), k_1_ (1/min) and, k_2_ (L/mol.min) are the rate constants of the pseudo-first-order and pseudo-second-order kinetic models, respectively.

**Figure 10:**
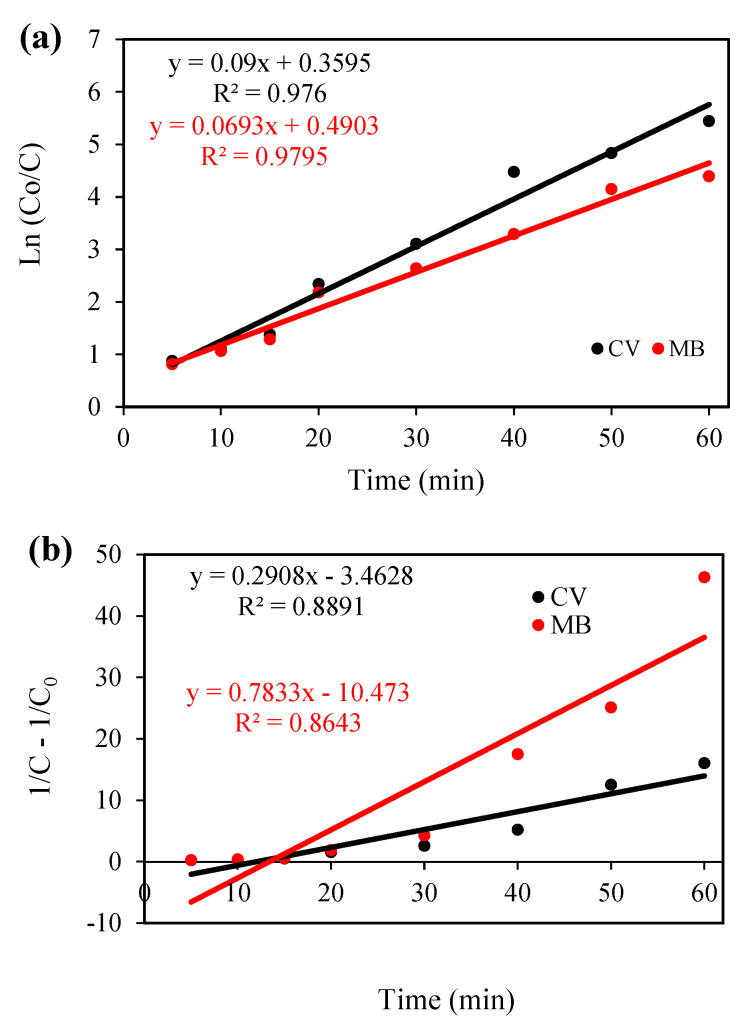
(**a**) Pseudo-first-order kinetic model and (**b**) pseudo-second-order kinetic model for the degradation of the desired dyes using the sono-photocatalytic process.

The linear relationship of the pseudo-first-order and pseudo-second-order kinetic models and the rate constant of the desired kinetic models for the simultaneous destruction process of CV and MB dyes are shown in Figure 10a,b, respectively. As the experimental results show, the correlation coefficient for the pseudo-first-order kinetic model is higher than the pseudo-second-order kinetic model, which shows that the kinetic behavior of the Cloisite 30B/ZnO/Ag_2_O sono-photocatalytic process in the simultaneous degradation of CV and MB cationic dyes follows the pseudo-first-order kinetic model. Additionally, the rate constant of the kinetic model for the degradation process of CV and MB dyes was determined to be 0.09 and 0.0693 min^−1^, respectively, which shows that the ability of the Cloisite 30B/ZnO/Ag_2_O sono-photocatalytic process in the degradation of CV dye is greater than that of MB dye. In Table 2, a comparison is made between the rate constant of the pseudo-first-order kinetic model of the Cloisite 30B/ZnO/Ag_2_O sono-photocatalytic process with other photocatalysts used in the degradation of CV and MB dyes, which shows that the Cloisite 30B/ZnO/Ag_2_O nanocomposite has a good ability regarding the simultaneous degradation of CV and MB dyes from aqueous solutions. Additionally, in this table, the effect of the studied catalyst components on the removal of dyes was investigated, which have a much lower efficiency compared to those of the original composite.

### 3.7. Proposed Mechanism for Dye Degradation Using Cloisite 30B/ZnO/Ag_2_O Photocatalyst

**Figure 11:**
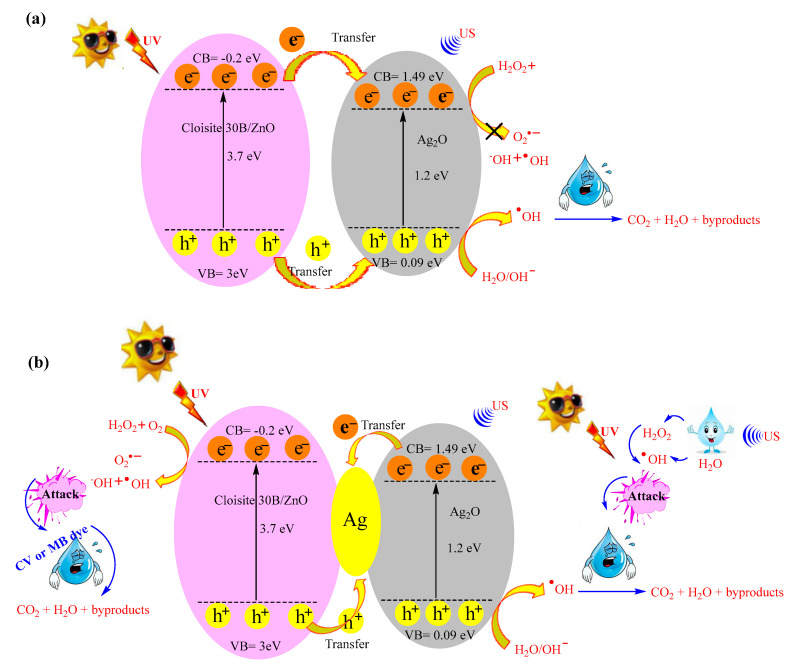
Proposed mechanism for dyes degradation using Cloisite 30B/ZnO/Ag_2_O photocatalyst, (**a**) the generated electron in the conduction band of Ag_2_O (CB) cannot react with oxygen to produce •O_2_^−^, (**b**) the electrons produced in the Cloisite 30B/ZnO segment and produce the active species ^•^O_2_^−^ and ^•^OH.

Based on the experimental results, a possible mechanism for the degradation of MB and CV dyes using Cloisite 30B/ZnO/Ag_2_O photocatalyst is proposed as shown in Figure 11. As the results showed, the Cloisite 30B/ZnO/Ag_2_O photocatalyst was successfully synthesized, and the components of the photocatalyst have a good relationship with each other, and the desired photocatalyst has a type I heterojunction structure [77]. As shown in Figure 11, the value of CB for ZnO and Ag_2_O nanoparticles is −0.2 eV and 1.49 eV, respectively, and the value of VB for ZnO and Ag_2_O nanoparticles is determined to be 3 eV and 0.09 eV, respectively. The connection of the photocatalyst components with each other causes the electrons excited by UV to be transferred from Cloisite 30B/ZnO nanoparticles to Ag_2_O through heterogeneous bonds, and the photoholes produced from Cloisite 30B/ZnO are also transferred to Ag_2_O nanoparticles [78]. However, the generated electron in the conduction band of Ag_2_O (CB) cannot react with oxygen to produce ^•^O_2_^−^, because Ag_2_O particles have a higher conduction band than O_2_/^•^O_2_^−^ (Figure 11a). Therefore, since the Cloisite 30B/ZnO/Ag_2_O photocatalyst does not have the ability to produce ^•^O_2_^−^, mechanism b, which was proposed in previous investigations of how to produce reactive active species using Cloisite 30B/ZnO/Ag2O photocatalyst completely, is presented [77]. According to Figure 11b, during the sono-photocatalytic process, dissolved oxygen in the reaction medium and H_2_O_2_ consume the electrons produced in the Cloisite 30B/ZnO segment and produce the active species ^•^O_2_^−^ and ^•^OH. At the same time, the holes produced in the Ag_2_O part react with hydroxide and water and produce the active species ^•^OH [79]. In addition, Ag_2_O in a heterogeneous reaction in the presence of H_2_O_2_ when exposed to ultrasonic waves, leads to the breakdown of H_2_O_2_ molecules and the production of the reactive active species •OH. Additionally, ultrasonic waves in the oxidation process cause the formation of hot spots with high temperature and pressure, which results in the formation of water molecules and reactive active species •OH and •H. These species can participate in the oxidation process of MB and CV molecules. In the following, for a correct understanding of the mechanisms and the role of the mentioned components in the structure of the Cloisite 30B/ZnO/Ag_2_O photocatalyst, equations are presented that show the mechanism of reactive active species well (Equations (10)–(18)).

(10) $$\mathrm{Catalyst}\;+\;h\nu \to {\mathrm{Catalyst}\;(e}_{\mathrm{CV}}^{-})+{\mathrm{Catalyst}\;(h}_{\mathrm{VB}}^{+})$$

(11) $${\mathrm{Catalyst}\;(e}_{\mathrm{CV}}^{-})+O_{2}\to •O_{2}^{-}\;$$

(12) $$•O_{2}^{-}+e^{-}+{2H}^{+}\to H_{2}O_{2}$$

(13) $$H_{2}O_{2}+e^{-}\to •\mathrm{OH}+{\mathrm{OH}}^{-}$$

(14) $${\mathrm{Catalyst}\;(h}_{\mathrm{VB}}^{+})+H_{2}O\to •\mathrm{OH}+H^{+}$$

(15) $$\mathrm{Catalyst}+\mathrm{Ultrasonic}+H_{2}O\to •\mathrm{OH}+•H$$

(16) $$•\mathrm{OH}+•\mathrm{OH}\to H_{2}O_{2}$$

(17) $$\mathrm{Catalyst}+\mathrm{Ultrasonic}+H_{2}O_{2}\to 2•\mathrm{OH}$$

(18) $$•\mathrm{OH}+\mathrm{dyes}\to {\mathrm{CO}}_{2}+H_{2}O+\mathrm{byproducts}$$

### 3.8. Textile Wastewater Treatment and Dye Fixation

The TOC and BOD parameters of a sample of textile wastewater and distilled water containing dyes are shown in Table 3. As the data show, about 56% of dyes are stabilized from distilled water, while for real wastewater, this amount has reached about 17%. The reason for this difference can be considered the presence of various radical consumers and complex compounds in real wastewater. The pH factor of both wastewater samples was slightly decreased to a neutral value, which indicates that the catalyst is probably amphoteric.

## 4. Conclusions

In summary, the new Cloisite 30B/ZnO/Ag_2_O photocatalyst was successfully synthesized using chemical and hydrothermal deposition methods and was used in the sono-photocatalytic process for the simultaneous degradation of MB and CV cationic dyes. The results of morphology and physical properties showed that the desired photocatalyst was successfully synthesized and has a suitable structure and active surface, and its components have a suitable and effective interaction with each other. In the simultaneous sono-photocatalytic process of CV and MB dyes, the effect of various parameters on the catalytic activity was investigated, and the results showed that the maximum simultaneous degradation efficiency of MB (98.43%) and CV (99.21%) dyes were at pH = 8, reaction time 50 min, H_2_O_2_ amount 40 mM, catalyst dose 0.5 g/L and the initial concentration of the dye pollutant 5 mg/L. Additionally, the results showed that the desired catalyst can be reused in the sono-photocatalytic process for up to 4 steps, and in the simultaneous degradation process of the desired dyes, reactive active species OH• and h^+^ have an effective role. The kinetic study showed that the sono-photocatalytic process of the degradation of CV and MB dyes followed the pseudo-first-order kinetic model, and the reaction rate constants for the degradation of CV and MB dyes were determined to be 0.09 and 0.0693 min^−1^, respectively. In addition, the toxicity study showed that the aqueous solution after the sono-photocatalytic process does not have any special toxicity and is not dangerous for aquatic animals and microorganisms, and can be used for various purposes, such as agriculture and aquaculture, or released into the environment.

## Figures and Tables

**Table 1:** The main compounds produced from the dye degradation using the sono-photocatalytic process.

The Main Intermediate	Content (µg)	The Main Intermediate	Content (µg)
Trans-1,2-DichloroeEthene	<0.05	1,1,2,2-TetrachloroEthane	<0.05
Cis-1,2-DiChloroEthane	<0.05	Isopropyle Benzene	<0.05
Chloroform	<0.05	1,2,3-Trichloropropane	<0.05
1,1,1-TriChloroEthane	<0.05	BromoBenzene	<0.05
1,2-DichloroEthane	<0.05	4-ChloroToluene	<0.05
Benzene	<0.05	n-propylBenzene	<0.05
1,2-DichloroPropane	<0.05	2-chloroToluene	<0.05
Dibromo Methane	<0.05	1,2,4TrimethylBenzene	<0.05
BromoDichloroMethane	<0.05	tert-ButylBenzene	<0.05
TRANS-1,3-DichloroPropene	<0.05	1,3,5,trimethylBenzene	<0.05
Toluene	<0.05	1,4-DichloroBenzene	<0.05
1,1,2-TrichloroEthane	<0.05	Sec-ButylBenzene	<0.05
1,3-Dichloro Propane	<0.05	1,3-DichloroBenzene	<0.05
Dibromochloro Methane	<0.05	p-Cymene	0.1
DibromoEthane	<0.05	1,2-DichloroBenzene	<0.05
ChloroBenzene	<0.05	n-ButylBenzene	<0.05
1,1,1,2-TetrachloroEthane	<0.05	1,2-Dibromo-3-ChloroPropane	<0.05
EthylBenzene	<0.05	1,2,3-TriChlorBenzene	<0.05
m-Xylene	<0.05	Naphtalene	<0.05
p-Xylene	<0.05	HexaChlorobutdiene	<0.05
o-Xylene	<0.05		

**Table 2:** Rate constant comparison of pseudo-first-order kinetic model and sono-photocatalytic performance of Cloisite 30B/ZnO/Ag_2_O with other photocatalysts used for dyes degradation.

Photocatalysts	Dye Content	Reaction Time/Photocatalyst Content	Power of the Light Source	Removal Efficiency (%)	Kinetic Rate Constant (1/min)	Ref.
SnO_2_	MB: 5 mg/L	40 min/----	7 W UV-LEDs	88.33	0.07	[70]
β-NiMoO_4_	MB: 4 mg/L	150 min/1 g/L	Diffused Sunlight	98.2	----	[71]
ZnO(70)/GO	MB: 20 mg/L	130 min/0.5 g/L	160 W Hg Lamp	93	0.01	[27]
Bmim[OAc]-Cu_2_O/g-C_3_N_4_	MB: 5 mg/L	30 min/0.1 g/L	6W UV-C lamp	100	0.151	[61]
TiO_2_/CuO/NGP	MB:20 mg/L	120 min/0.3 g/L	40 W UV-C/40 W Xe Lamp	----	0.09863	[72]
Ce-MoO_3_	MB: 5 mg/L	50 min/0.5 g/L	500 W Xe Lamp	65.5%	0.0171	[73]
α-Fe_2_O_3_/Bi_2_MoO_6_	MB:20 mg/L	240 min/2.5 g/L	500W Xe Lamp	90.7	----	[74]
F-TiO_2_(B)/fullerene	CV: 30 mg/L	120 min/0.1 g/L	UV Filter	82.93%	----	[61]
CTO	CV: 5 mg/L	60 min/10 g/L	Solar	37.24	0.0002	[75]
CTO@S	CV: 5 mg/L	60 min/10 g/L	Solar	89	0.008	[75]
ZnTiO_3_@S NC	CV: 10 mg/L	60 min/1 g/L	Natural Sunlight	87.81	0.0092	[76]
Closite 30B/ZnO/Ag_2_O	CV: 5 mg/L	50 min/0.5 g/L	12 W UV Lamp	99.21	0.09	This study
Closite 30B/ZnO/Ag_2_O	MB: 5 mg/L	50 min/0.5 g/L	12 W UV Lamp	98.43	0.0693	This study
Closite 30B	CV: 5 mg/L	50 min/0.5 g/L	12 W UV Lamp	16%	0.02 ^a^	This study
Closite 30B	MB: 5 mg/L	50 min/0.5 g/L	12 W UV Lamp	15%	0.02 ^a^	This study
ZnO	CV: 5 mg/L	50 min/0.5 g/L	12 W UV Lamp	25%	0.031	This study
ZnO	MB: 5 mg/L	50 min/0.5 g/L	12 W UV Lamp	25%	0.030	This study
Ag_2_O	CV: 5 mg/L	50 min/0.5 g/L	12 W UV Lamp	36%	0.042	This study
Ag_2_O	MB: 5 mg/L	50 min/0.5 g/L	12 W UV Lamp	35%	0.041	This study

^a^ R^2^ value was <0.7.

**Table 3:** Characteristics of the textile and synthetic wastewater (catalyst dose: 0.5 g/L, reaction time: 50 min, amount of H_2_O_2_: 40 mM/100 mL, and pH = 8).

Sample	Parameters Value							
	pH		COD (mg/L)		BOD_5_ (mg/L)		TOC (mg/L)	
	Fresh	Treated	Fresh	Treated	Fresh	Treated	Fresh	Treated
Distilled water + MB ^1^	8.0	7.8	12	7	26	5	18	7.5
Distilled water + +CV ^1^	8.0	7.8	13	7.8	28	5	19	8.3
Textile wastewater	7.5	7.41	1305	823	639	330	397	329

^1^ Dye concentration: 5 mg/L.

## Data Availability

Not applicable.
